# Physiological and Yield Responses of Pepper (*Capsicum annuum* L.) Genotypes to Drought Stress

**DOI:** 10.3390/plants14131934

**Published:** 2025-06-24

**Authors:** Theodora Ntanasi, Ioannis Karavidas, Dimitrios Savvas, George P. Spyrou, Evangelos Giannothanasis, Beppe Benedetto Consentino, Vasileios Papasotiropoulos, Leo Sabatino, Georgia Ntatsi

**Affiliations:** 1 Laboratory of Vegetable Production, Department of Crop Science, Agricultural University of Athens, Iera Odos 75, 11855 Athens, Greece; ntanasi@aua.gr (T.N.); karavidas@aua.gr (I.K.); dsavvas@aua.gr (D.S.); gspyrou@aua.gr (G.P.S.); giannothanasis@aua.gr (E.G.); 2 Department of Agricultural, Food and Forest Sciences, University of Palermo, 90128 Palermo, Italy; beppebenedetto.consentino@unipa.it (B.B.C.); leo.sabatino@unipa.it (L.S.); 3 Laboratory of Plant Breeding and Biometry, Department of Crop Science, Agricultural University of Athens, Iera Odos 75, 11855 Athens, Greece; vpapasot@aua.gr

**Keywords:** *Capsicum annuum* L., abiotic stress, productivity, quality, nutrient concentration, soilless culture, osmotic stress

## Abstract

Drought stress is a critical abiotic constraint on agricultural productivity, particularly affecting crops like pepper (*Capsicum annuum* L.), which are highly susceptible to water deficits due to their physiological characteristics. The present study investigated the impact of a 40% reduction in irrigation on yield, macronutrient concentrations, and fruit quality across several pepper genotypes. The cultivars evaluated included two landraces, namely ‘JO109’ and ‘JO204’ (*Capsicum annuum* var. *grossum*), as well as the California cultivar ‘Yolo Wonder’ and the commercial F1 hybrid ‘Sammy RZ’, which served as controls. The experiment was conducted at the greenhouse facilities of the Laboratory of Vegetable Production, Agricultural University of Athens. Under reduced irrigation, most of the cultivars studied exhibited a decline in yield, which was attributed to a decrease in fruit number in ‘Yolo Wonder’ and a reduction in fruit weight in both ‘JO204’ and ‘Sammy’. In contrast, the landrace ‘JO109’ exhibited consistent yields under both growing conditions, a response likely attributed to elevated K concentration in the leaves and lower Na accumulation in the fruit, indicating enhanced tolerance to water deficit. A decline in leaf K concentration was observed in response to drought stress, while concomitantly increased concentrations of Na, Ca and Mg were recorded. Among fruit macronutrients, only Ca showed a significant decrease under reduced irrigation. Furthermore, fruit firmness (FF), titratable acidity (TA) and total soluble solids content (TSSC) exhibited higher levels under drought stress, particularly in ‘JO109’, while TA remained unaltered. These findings highlight the potential of landraces such as ‘JO109’ to be utilized in breeding programs aimed at enhancing resilience, while maintaining pepper fruit quality under limited water availability.

## 1. Introduction

The long-term effects of global climate change are expected to exacerbate abiotic stresses, leading to more frequent and prolonged droughts, elevated temperatures, and unpredictable rainfall patterns across many regions of the world [1]. A global assessment conducted by the Food and Agriculture Organization [2] projected that feeding a growing global population would require approximately 70% more food production by 2050. In the Mediterranean region, water availability is of particular concern, as both human activity and ongoing climate change have led to an intensification of drought conditions [3]. It is well established that evident that drought stress leads to substantial reduction in plant growth, primarily due to the disruption of vital processes such as cell division and cell expansion. These processes are governed by complex interactions among anatomical, physiological, and genetic factors. These interactions are adversely affected by limited water availability [4]. Drought is recognized as one of the most critical abiotic stresses affecting agricultural productivity, with the potential to reduce average crop yields by over 50% [5,6].

Drought stress poses a significant challenge to the growth, development, and yield of vegetable crops, as water accounts for more than 90% of the fresh weight of most vegetables [7,8]. It is well documented that vegetables typically exhibit sensitivity to drought when water content reaches approximately 20% of their biomass [9]. Water deficiency directly hinders photosynthesis by limiting CO_2_ diffusion into the drought stressed leaves due to reduced conductance, primarily in the stomatal and mesophyll tissues [10]. Furthermore, in conditions of drought, the uptake of nutrients by roots is reduced due to decreased soil water content, which slows the diffusion of nutrients from the soil to the root surface [11]. In addition to its adverse effects on plant growth, drought stress can influence the quality of the harvested fruits. Research on hydroponically grown vegetable crops under water limited conditions has shown that such stress can increase the concentration of sugars and acids in fruits, potentially improving their nutritional value, flavor, and taste [12].

Pepper (*Capsicum annuum* L.) is a key crop cultivated in Mediterranean regions, where limited water availability poses a significant challenge to its production [13]. The primary appeal of pepper is attributable to its flavour, nutritional content, and contribution to a healthy diet [14]. Bell pepper is highly sensitive to drought stress, primarily due to its extensive leaf surface area and elevated stomatal conductance [15]. It has been demonstrated that the occurrence of drought stress during the early developmental and reproductive phases leads to a decline in both the quantity and size of buds and fruits [16]. Furthermore, drought stress significantly impacts the yield of harvested pepper fruits [17]. The increased need for drought tolerant pepper varieties that can withstand reduced water availability, while maintaining acceptable yields and fruit quality, is evident by numerous studies [18]. Therefore, in agroecological and organic farming systems, maintaining a diverse range of crop varieties—particularly traditional and locally adapted ones that can be effectively used for breeding and as rootstocks—is predicted to enhance the resilience of vegetable production in the face of increasingly unpredictable future weather patterns [19,20].

Landraces frequently manifest as components of a more extensive metapopulation dispersed across landscapes exhibiting diverse environmental conditions, including variations in water availability [21]. However, it should be noted that these varieties may also be subject to certain limitations, including extended maturation times and relatively low yields. [22]. Notwithstanding the aforementioned disadvantages, landraces are considered to be of significant value as genetic resources for the purpose of crop improvement, particularly with regard to traits associated with quality [23]. It is evident that local landraces have adapted to specific environments, thus demonstrating the potential to exhibit superior performance in comparison to non-local populations under local conditions. This phenomenon can be attributed to the evolutionary selection of particular traits in response to their respective environments [24].

To address this, the present study aimed to assess the performance of various pepper cultivars—either native to or commonly grown in the Mediterranean region—under conditions of limited irrigation. Our evaluation focused on yield, fruit quality, and the nutritional status of both leaves and fruits. A thorough analysis of the findings will help identify traits associated with drought tolerance in pepper plants. This information can support breeding initiatives aimed at developing (a) new varieties or hybrids capable of thriving in drought—prone environments without compromising yield, or (b) improved rootstocks for grafted plant production. The novelty of this study lies in the use of a fully controlled open hydroponic system to apply precise water stress treatments. This allows the differentiation of drought responses and the identification of agronomic and quality characteristics unique to each Mediterranean landrace.

## 2. Results

**Figure 1:**
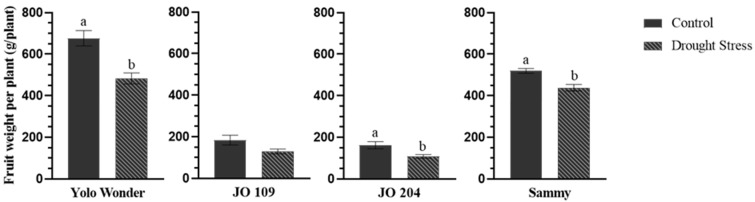
Effect of drought stress on fruit weight per plant (g/plant) across different pepper genotypes. The label ‘Control’ represents well-irrigated conditions, while ‘Drought stress’ indicates exposure to limited water availability (40% reduction). Different letters above each bar signify statistically significant differences based on Duncan’s multiple range test (*p* < 0.05). Vertical bars represent the standard error of the mean (n = 4).

The yield of different pepper genotypes grown under either normal or reduced irrigation is illustrated in Figure 1. The commercial variety ‘Yolo Wonder’ and the hybrid ‘Sammy’ were the most productive, exhibiting a high yield per plant. However, under water stress conditions, their yield declined by 28% and 15%, respectively. A more pronounced decline, reaching 33%, was observed in the landrace ‘JO204’. In contrast, the traditional Jordanian landrace ‘JO109’ did not show a statistically significant yield reduction under limited water availability.

As shown in Table 1, the decline in yield observed for the varieties ‘JO204’ and ‘Sammy’ under drought stress, was attributed to a reduction in fruit weight rather than fruit number. Specifically, average fruit weight decreased by 28% in ‘JO204’ and by 9% in ‘Sammy’. In contrast, the yield reduction in the variety ‘Yolo Wonder’ under water shortage conditions for was associated with a 23% decrease in fruit number.

Table 2 shows the concentrations of the macronutrients potassium (K), sodium (Na), calcium (Ca) and magnesium (Mg) in the leaves of different pepper cultivars. Water stress significantly affected all four nutrients. Specifically, K increased by 12% under drought conditions, while the concentrations of Na, K/Na, Ca, and Mg decreased by 16%, 24%, 17%, and 14%, respectively. There was significant variation among cultivars in the concentrations of K, Na, and Ca, whereas no significant differences were observed for Mg. The cultivar ‘JO109’ demonstrated the highest concentrations of K, K/Na and Na in its leaves, and together with ‘JO204’, the lowest Ca content. Moreover, a significant interaction between drought stress and cultivar was found only for K. It is noteworthy that K concentration in ‘JO204’ leaves remained unaffected under drought stress.

Table 3 presents the concentrations of the macronutrients K, Na, Ca and Mg in pepper fruits. Drought stress showed a significant decrease of 14% on Ca levels, compared to the control conditions. In contrast, significant differences among pepper cultivars were observed for all four macronutrients. Specifically, the cultivar ‘JO204’ exhibited the highest concentrations of K, Na, K/Na and Mg, and the lowest of Ca. ‘JO109’ exhibited the lowest Na concentration and the highest K/Na ratio and Mg concentration in its fruits. The cultivars ‘Yolo Wonder’ and ‘Sammy’ exhibited the highest Na concentrations in fruit tissue. A significant interaction was identified between the effects of drought stress and the variety for both Na and Ca, with ‘JO204’ displaying notable changes, including a 55% increase in fruit Na concentration and a 40% decrease in Ca concentration under drought conditions.

**Figure 2:**
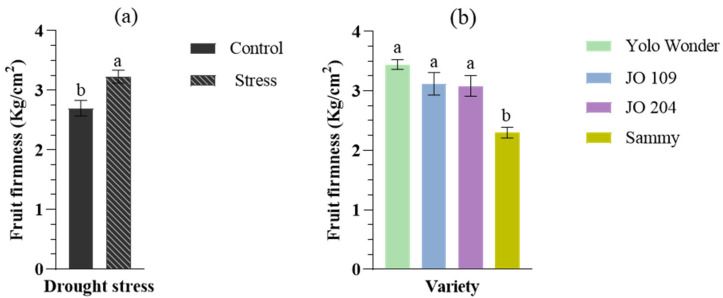
Effect of drought stress (**a**) and genotype (**b**) on fruit firmness in different cultivated pepper genotypes. (**a**) ‘Control’ refers to well-irrigated conditions, while ‘Stress’ indicates exposure to limited water availability. Genotype names are provided in the legend (**b**). Different letters above bars indicate statistically significant differences according to Duncan’s multiple range test (*p* < 0.05). Error bars represent the standard error of the mean (n = 10).

Fruit firmness was significantly increased (by 20%) under drought stress (Figure 2). Statistically significant differences were also found among the different pepper cultivars. The varieties ‘Yolo Wonder’, ‘JO109’, and ‘JO204’ exhibited similar firmness values, while the ‘Sammy’ hybrid showed the lowest fruit firmness compared to the other cultivars. However, no statistical significance was observed in the interaction of the two factors (drought × variety).

**Figure 3:**
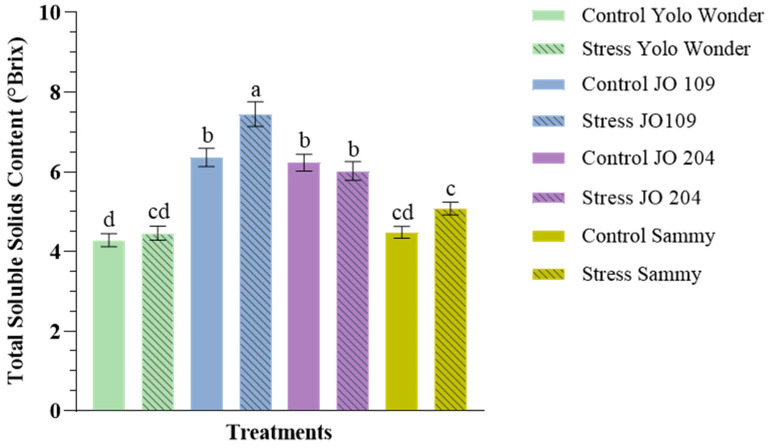
Impact of drought stress and genotype on the total soluble solid content in pepper fruits. Different letters above the bars indicate statistically significant differences among treatments based on Duncan’s multiple range test (*p* < 0.05). Error bars represent the standard error of the mean (n = 10).

Regarding total soluble solids content (TSSC), both drought stress and cultivar had a significant effect, with TSSC increasing by 6% under drought conditions (Table 4). Among the cultivars, ‘JO109’ exhibited the highest value for TSSC, while the commercial variety ‘Yolo Wonder’ recorded the lowest. A significant interaction between drought stress and variety was also observed (Figure 3). Notably ‘JO109’, which had the highest TSSC among all varieties, exhibited a 17% increase in TSSC under drought conditions, the greatest increase recorded among the tested cultivars.

**Figure 4:**
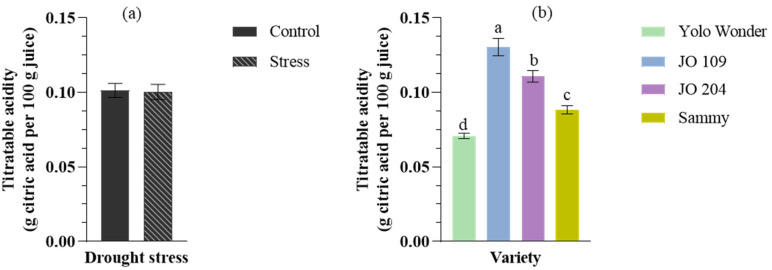
Effect of drought stress (**a**) and genotype (**b**) on fruit titratable acidity in different pepper cultivars. (**a**) ‘Control’ refers to well-watered conditions, while ‘Stress’ indicates limited water availability. (**b**) Genotype names are provided in the legend. Different letters above the lines indicate statistically significant differences according to Duncan’s multiple range test (*p* < 0.05). Error bars represent the standard error of the mean (n = 10).

Finally, statistically significant differences were observed for titratable acidity (TA) only among the cultivated varieties (Figure 4). Specifically, the landrace ‘JO109’ exhibited the highest TA value, while the commercial variety ‘Yolo Wonder’ exhibited the lowest. Neither drought stress nor the interaction drought × variety showed any statistically significant effect on TA.

## 3. Discussion

Drought stress is a well-studied abiotic factor that has been demonstrated to substantially limit crop growth and yield [25]. According to Earl et al. [26], the reduction in yield under water stress conditions is primarily associated to decreased light interception, reduced photosynthetic efficiency, lower water use efficiency (WUE), and a decline in the harvest index. Pepper plants exhibit increased sensitivity to water shortage, primarily attributed to their pronounced leaf surface area and elevated stomatal conductance, which amplify water loss during periods of drought [15]. In the present study, a 40% reduction in irrigation compared to well-watered plants led to a noticeable decrease in yield, with some varieties exhibiting reductions of up to 20–25%. Comparable findings were reported by Dorji et al. [27], who observed a 35% reduction in hot pepper yield when irrigation was decreased to 50% of normal levels. Furthermore, as demonstrated in numerous studies on drought stress in different crops [6,28,29], yield losses are frequently attributed to the suppression of plant growth resulting from decreased photosynthetic activity, reduced chlorophyll content in leaves, and limited nutrient and water uptake by the roots due to inadequate irrigation.

In the present study, the yield reduction under drought conditions was attributed to a decrease in fruit number in the bell pepper cultivar ‘Yolo Wonder’, whereas in cultivars ‘JO 204’ and ‘Sammy’, it was primarily due to a reduction in the mean fruit weight. Similar findings were documented by Ficiciyan et al. [17], who noted a 10% decline in the yield of sweet pepper fruits subjected to drought stress conditions. In a study conducted by Kurunc et al. [30], it was observed that the mean fresh fruit weight of long peppers was reduced under water deficit, as was also the case in the varieties examined in the present study. However, the ‘JO109’ genotype exhibited resilience to drought stress, as evidenced by its stable yield performance under water-limited conditions—a trait presumably associated with its landrace origin. A similar observation was reported by Fullana-Pericàs et al. [31] in their study on tomato cultivars, where several local varieties demonstrated comparable or even superior performance to modern ones in mitigating yield reduction under drought stress. Concurrently, Techawongstien et al. [32] investigated the long-term effects of short-term water stress on key physiological traits at the pre-anthesis stage in four chilli pepper cultivars. They reported that genotypes exhibiting higher leaf water potential demonstrated greater drought tolerance, as evidenced by their ability to swiftly regain photosynthetic capacity upon rehydration, thereby minimizing the adverse effect on final fruit yield.

Drought stress significantly impairs the uptake of nutrients by roots and their transport to the shoots, primarily by restricting potassium (K) diffusion in the soil toward the root zone, which in turn limits K absorption [33]. As for the concentration of macronutrients (K, Na, Ca, Mg) in the leaves, it was observed that drought conditions resulted in a reduction in potassium levels in pepper plants. This finding is consistent with previous studies, such as Kirnak et al. [34], which reported reduced K concentration in leaves under water deficit conditions. In a similar study, Sadak et al. [35], observed a decline in K concentration of approximately 18% in the leaves of drought stressed pepper seedlings in comparison to the control plants. Among the different varieties tested, the ‘JO109’ landrace exhibited the highest K concentration in its leaves under drought stress conditions, despite the decline in leaf K levels across all varieties. The absence of yield reduction in the ‘JO109’ may be explained by its higher K levels, as K is known to alleviate various stresses experienced by plants, including drought, freezing, and high light intensity [36]. Conversely, K deficiency has been shown to disrupt CO_2_ assimilation, transport, and utilization processes during photosynthesis. [36]. The consistent yield of the landrace ‘JO109’ could be attributed to a high K/Na ratio in the leaves. As Hu and Schmidhalter [37] has demonstrated, the high K/Na ratio is also subject to regulation by the roots, indicating a selective uptake of K over Na and a preferential transport of K into the xylem. Concerning the remaining macronutrients, Na, Ca, and Mg, an increase in their concentrations was observed in pepper leaves under reduced irrigation conditions. Consistent findings were reported by Delfine et al. [38], who observed elevated levels of Ca and Na in drought stressed pepper plants in comparison to those under regular irrigation. Moreover, higher Na concentrations were observed in the leaves of landraces ‘JO109’ and ‘JO204’, whereas the commercial varieties, such as ‘Yolo Wonder’ and ‘Sammy’, exhibited the highest Ca concentrations. Similar findings were reported for these cultivars in the study by Ntanasi et al. [39].

In fruits, significant variations in the concentrations of K, Na, Ca and Mg macronutrients were observed among the different pepper varieties. However, reduced irrigation significantly affected only the Ca concentration, which exhibited a substantial decrease in pepper fruits. Similar findings were also reported by Martins et al. [40], who observed a 13.2% reduction in fruit Ca concentration under drought conditions in green peppers. No significant differences were observed for the other macronutrients (K, Na), a finding consistent with the study by De Pascale et al. [41]. Furthermore, the studies conducted by Birgin et al. [42] and De Pascale et al. [41] revealed no significant differences in Mg fruit concentration between drought-stressed and control plants. However, a notable observation from the present study is that, despite varietal differences in all measured macronutrients, the only variety that maintained stable yield under reduced irrigation conditions (JO109) was also the one that maintained a stable fruit K/Na under drought stress. In addition, this landrace possessed the lowest Na concentration in its fruits, a condition not observed in the leaves. Conversely, the remaining varieties demonstrated comparable Na levels in their fruit.

A number of studies have indicated that water deficit conditions may positively influence fruit quality by activating primary and secondary metabolic pathways [43]. Regarding fruit quality traits, reduced irrigation levels in pepper plants led to a substantial increase of fruit firmness compared to those cultivated under standard irrigation. This improvement may be attributed to notable differences in Ca concentration between the two treatments, reinforcing the established correlation between Ca levels and fruit firmness [44]. However, unlike the findings of Abdelkhalik et al. [45], who investigated the effects of varying deficit irrigation levels on sweet pepper, thid study did not observe any statistically significant differences in fruit firmness. Furthermore, a reduction in irrigation had a significant impact on total soluble solids content (TSSC), which increased under drought stress. Notably, varietal disparities in TSSC were recorded, with the ‘JO109’ landrace exhibiting the highest value. This landrace exhibited a substantial increase in TSSC under reduced irrigation compared to well-watered conditions, and was the only one that did not experience a decline in yield. These findings are consistent with those reported in previous research. For instance, Abdelkhalik et al. [45] documented a 26% increase in °Brix—which is closely related to TSSC- in sweet pepper when irrigation was reduced by 50% of the crop’s water requirements. Similarly, Maduranga Bandara Rathnayaka et al. [46] observed elevated °Brix levels in chili peppers under drought stress, with irrigation reduced by approximately 60%. Demir et al. [47] also reported significant increases in °Brix of pepper fruit, with values rising by approximately 10% under 75% irrigation and 16% under 50% of water requirements. The observed increase in TSSC under limited irrigation may be attributed to a reduced water accumulation in the fruit [48]. One possible explanation for this phenomenon is that when fruit development is hindered, the sugars produced through photosynthesis are distributed among fewer sinks, thereby increasing their concentration in individual fruits [49]. Additionally, Renquist et al. [50] proposed that the elevated soluble solids content observed under water-deficit conditions is a result of reduced water uptake, which leads to a concentration effect within the tomato fruit. However, no significant effect on TA was observed in pepper fruits, which is consistent with the findings of Abdelkhalik et al. [45], who reported similar results in sweet pepper under 50% water shortage of the plant’s requirements. In contrast, Demir et al. [47] reported a 5% increase in TA under a 50% reduction in irrigation compared to full irrigation, while no notable difference was observed when irrigation was reduced to 75% of normal levels.

## 4. Materials and Methods

### 4.1. Experimental Design

The experiment was carried out in the glasshouse facilities of the Laboratory of Vegetable Production at the Agricultural University of Athens (AUA), situated at 37°59′2″ N and 23°42′19″ E. The study comprised two Jordanian landraces, named ‘JO 109’ and ‘JO 204’, along with the ‘Yolo Wonder’ commercial variety and the ‘Sammy RZ’ hybrid, which were used as reference. Table 5 provides a comprehensive overview of the names and origins of the pepper seeds utilized in the experiment. The cultivation took place in an open soilless system with two irrigation levels, and the experimental design was based on a randomized complete block design (RCBD).

### 4.2. Growing the Seedlings

The seedlings were produced by the Laboratory of Vegetable Production at the Agricultural University of Athens. To ensure the viability of the initial material, all seeds underwent an initial disinfection process on 20 September. This process involved soaking the seeds in a 15% *v*/*w* solution of Na_3_PO_4_ for 20 min. Following disinfection, they were placed in Petri dishes and incubated in a temperature-controlled chamber at 25 °C for 5 days. On 27 September, the germinated seeds were transferred to sowing trays, using turf as a substrate, enabling seedlings growth.

### 4.3. Cultivation Practice

On November 13, the seedlings that had reached the stage of four or five true leaves were transplanted into 33-L perlite bags, thus commencing cultivation in an open hydroponic system in perlite bags that had been previously soaked to saturation with a starter solution (Table 2) for 24 h. At this stage, the application of a reduced nutrient solution flow rate was initiated. Specifically, the first treatment was applied to the control, i.e., plants receiving the recommended amount of irrigation, and the second treatment was applied to plants that were irrigated with reduced amounts, with irrigation requirements reduced by up to 40% compared to well-irrigated plants. This percentage was chosen because it represents the moderate level of stress that plants [51,52] commonly encounter in Mediterranean environments. This level of stress enables the plants to grow and produce fruit, which allows us to evaluate the effects of water stress. After dipping the perlite bags in the starter solution for 24 h, the bottom of each bag was perforated to allow proper drainage of excess nutrient solution. Each treatment was repeated four times, with four bags of perlite utilized for each treatment. Three plants of the same variety were placed in each bag of perlite. Throughout the growing season, the average temperatures maintained were 21 °C during the day and 17 °C during the night.

### 4.4. Nutrient Solution Formula

The formulation of the nutrient solution required for the cultivation of pepper crop was conducted using a specially designed computer tool, known as NUTRISENSE, accessible at https://nutrisense.online/ (accessed on 23 June 2025). This program utilizes advanced algorithms to accurately determine the required quantities of macro- and micronutrients, taking into consideration the specific growth stage of the plant, the current season, and the type of cultivation system employed. Prior to the transplantation of the pepper crop, a starter solution was applied to the growing substrate. During cultivation, two distinct nutrient solutions were prepared, customized to align with the vegetative and reproductive phases of plant development, respectively. The nutrient solutions dispensed to the plants were initially prepared by formulating concentrated stock solutions, which were then diluted to a ratio of 1:100. In the experiment, all plants were provided with the same standard nutrient solution, while the amount of irrigation varied. Half of the plants received the recommended amount of water, thus serving as the control group. The remaining half were subjected to deficit irrigation, receiving up to 40% less NS than the well-irrigated control plants. The pH of the nutrient solution was meticulously adjusted daily to 5.6, employing an appropriate volume of 1 N HNO_3_ solution. The specific concentrations of macro- and micronutrients for each treatment and growth stage are presented in Table 6.

### 4.5. Sampling of Leaves and Fruits for Macro- and Micronutrient Evaluation

At the final stage of the experiment, samples were collected from pepper plants from all treatments. The third, fourth and fifth leaves were collected from the uppermost portion of each plant. Furthermore, fruit samples were collected for analysis. Four replicates per treatment were used for nutrient analyses. Subsequently, both leaf and fruit samples were dried at 65 °C until a constant weight was achieved, ensuring consistency.

### 4.6. Assessment of Crop Yield

Τhe first fruit harvest was conducted on 11 March, while the final harvest took place on 7 April. Harvesting was carried out approximately once or twice weekly, and the fruits harvested were selected based on the criterion of achieving commercial maturity. The process involved manual collection or the use of scissors, ensuring that the fruits were removed from the plant along with their calyx and a portion of the pedicel. Following each harvest, a meticulous enumeration and weighing of the fruits was conducted. The data collection process encompassed various parameters, including the total number of fruits per plant, the total fresh weight of fruit per plant (g plant^−1^), and the average fresh weight of individual fruit (g).

### 4.7. Nutrient Content in Leaves and Fruit

After the drying process, all samples were ground using an MF 10 Basic Micro Fine Grinder (IKA Werke, Staufen, Germany). The extraction process was performed through the dry ashing method, and the potassium (K) and sodium (Na) levels were assessed using a flame photometer (Sherwood Model 410, Cambridge, UK). Concentrations of calcium (Ca) and magnesium (Mg) were measured using an atomic absorption spectrophotometer (AA-7000, Shimadzu Co., Tokyo, Japan).

### 4.8. Organoleptic Characteristics

Quality attributes, including titratable acidity (TA), total soluble solids content (TSSC) and fruit firmness (FF), were evaluated using ten ripe fruits per treatment. Fruit acidity (FA) was analyzed by potentiometric titration, where 10 mL of juice was titrated with 0.02 M NaOH until reaching a pH of 8.1. Firmness was assessed with a Mechanical Force Gauge (Chatillon penetrometer—DPP5KG).

### 4.9. Statistical Analysis

In this study, one-way analysis of variance (ANOVA) was conducted to assess the primary effects of water stress on yield. Furthermore, a two-way ANOVA was conducted to assess the organoleptic characteristics and nutrient composition in plant tissues across different pepper cultivars. The statistical analysis was conducted using the STATISTICA software package (version 12.0 for Windows). In instances where drought stress exerted a significant influence on a measured parameter in the cultivars, mean comparisons were conducted employing the Duncan’s Multiple Range Test at a significance level of *p* ≤ 0.05.

## 5. Conclusions

The present study confirms the significant impact of drought stress on pepper (*Capsicum annuum* L.) yield, nutrient uptake, and fruit quality, highlighting the varying degrees of sensitivity among different cultivars. A 40% reduction in irrigation resulted in substantial yield losses across most cultivars, primarily attributable to a decline in fruit number or size. However, the landrace ‘JO109’ exhibited notable resilience, maintaining stable yields under water limited conditions. This tolerance appears to be linked to the higher leaf K concentration and lower Na concentration in fruit, reinforcing the crucial role of K in mitigating drought induced physiological stress. In relation to fruit quality, water deficit conditions were found to enhance TSSC and FF, particularly in ‘JO109’, without exerting a negative effect on TA. These enhancements in quality are presumably associated with physiological adjustments that concentrate solutes due to reduced water content and altered metabolic activity under stress. The findings of this study highlight the potential of landraces, such as ‘JO109’, for incorporation into breeding programs that are focused on enhancing drought resilience and fruit quality in pepper.

## Figures and Tables

**Table 1:** Effect of drought stress on the average fruit number per plant (FN) and the mean fruit weight (MFW) across the tested genotypes.

	FN (No Plant^−1^)				MFW (g)			
Water Availability	Yolo Wonder	JO109	JO204	Sammy	Yolo Wonder	JO109	JO204	Sammy
Control	4.75	14.17	13.88	12.08	142.22	12.81	11.61	43.00
Drought stress	3.67	10.92	13.00	11.17	132.29	11.96	8.31	39.33
Statistical significance	**	NS	NS	NS	NS	NS	**	*

Mean values (n = 4) represent statistically significant differences according to Duncan’s multiple range test (*p* < 0.05). Asterisks indicate significance levels: ** (*p* < 0.01), and * (*p* < 0.05), while NS signifies a non-significant difference.

**Table 2:** Effect of drought stress and cultivar on leaf macronutrient concentration in the tested genotypes.

Leaves						
Water Availability	Variety	K (mg/g)	Na (mg/g)	K/Na	Ca (mg/g)	Mg (mg/g)
Main effects (Drought stress)						
Control		43.75	0.25	176.06	30.04	9.20
Drought stress		38.38	0.29	134.47	35.15	10.48
Main effects (Variety)						
	Yolo Wonder	38.75 b	0.23 c	171.32 a	35.34 a	10.73
	JO 109	52.00 a	0.33 a	166.86 a	27.10 b	9.40
	JO 204	39.50 b	0.29 ab	140.03 b	30.03 b	9.58
	Sammy	34.00 c	0.25 bc	142.84 b	37.91 a	9.64
Interaction						
Control	Yolo Wonder	41.50 c	0.23	180.68 ab	33.27	10.52
	JO 109	55.00 a	0.29	197.30 a	24.23	8.48
	JO 204	39.00 cd	0.26	148.97 bc	27.13	8.36
	Sammy	39.50 cd	0.23	177.29 ab	35.54	9.42
Drought stress	Yolo Wonder	36.00 d	0.23	161.96 bc	37.40	10.94
	JO 109	49.00 b	0.38	136.43 cd	29.96	10.33
	JO 204	40.00 cd	0.31	131.10 cd	32.93	10.79
	Sammy	28.50 e	0.27	108.39 d	40.29	9.86
Statistical significance						
Drought Stress		***	**	***	***	***
Variety		***	***	**	***	NS
Drought Stress × Variety		**	NS	*	NS	NS

Mean values (n = 4) followed by different letters within the same column represent statistically significant differences according to Duncan’s multiple range test (*p* < 0.05). Asterisks indicate significance levels: *** (*p* < 0.001), ** (*p* < 0.01) and * (*p* < 0.05), while NS signifies a non-significant difference.

**Table 3:** Effect of drought stress and cultivar on fruit macronutrient concentration in the tested genotypes.

Fruit						
Drought Stress	Variety	K (mg/g)	Na (mg/g)	K/Na	Ca (mg/g)	Mg (mg/g)
Main effects (Drought stress)						
Control		47.88	0.18	279.56	11.28	5.59
Drought stress		49.75	0.19	269.59	9.66	5.58
Main effects (Variety)						
	Yolo Wonder	45.25 c	0.21 a	226.47 b	10.91 b	5.15 b
	JO 109	49.50 b	0.15 b	341.04 a	9.33 c	5.96 a
	JO 204	59.25 a	0.20 a	306.26 a	9.09 c	6.23 a
	Sammy	41.25 d	0.19 a	224.54 b	12.56 a	4.99 b
Interaction						
Control	Yolo Wonder	43.00	0.22 ab	204.65 b	11.35 abc	5.27
	JO 109	49.00	0.15 cd	322.41 a	9.29 c	6.07
	JO 204	57.50	0.16 cd	358.69 a	11.37 abc	5.88
	Sammy	42.00	0.19 bcd	232.50 b	13.11 a	5.13
Drought stress	Yolo Wonder	47.50	0.20 abc	248.30 b	10.46 bc	5.02
	JO 109	50.00	0.14 d	359.67 a	9.37 c	5.85
	JO 204	61.00	0.25 a	253.83 b	6.82 d	6.58
	Sammy	40.50	0.19 bcd	216.58 b	12.01 ab	4.85
Statistical significance						
Drought Stress		NS	NS	NS	**	NS
Variety		***	**	***	***	***
Drought Stress × Variety		NS	**	**	**	NS

Mean values (n = 4) followed by different letters within the same column represent statistically significant differences according to Duncan’s multiple range test (*p* < 0.05). Asterisks indicate significance levels: *** (*p* < 0.001) and ** (*p* < 0.01), while NS signifies a non-significant difference.

**Table 4:** Impact of drought stress and cultivar on the total soluble solids content (TSSC) in the fruits of the evaluated pepper genotypes.

Water Stress	Variety	TSSC (°Brix)
Main effects (Drought stress)		
Control		5.41
Drought stress		5.74
Main effects (Variety)		
	Yolo Wonder	4.37 d
	JO 109	6.81 a
	JO 204	6.12 b
	Sammy	4.81 c
Statistical significance		
Drought Stress		**
Variety		***
Drought Stress × Variety		*

Mean values (n = 10) followed by different letters within the same column represent statistically significant differences according to Duncan’s multiple range test (*p* < 0.05). Asterisks indicate significance levels: *** (*p* < 0.001), ** (*p* < 0.01) and * (*p* < 0.05).

**Table 5:** Details on the origins and names of the pepper seeds cultivated.

Variety	Type	Provider
Yolo Wonder	Reference	INRA ^1^
JO109 (*Capsicum anuum* var. *grossum*)	Landrace	NARC ^2^
JO204 (*Capsicum anuum* var. *grossum*)	Landrace	NARC ^2^
Sammy RZ (F1-Hybrid)	Reference	Rijk Zwaan ^3^

^1^ Institut National de la Recherche Agronomique ^2^ The National Agricultural Research Center ^3^ Vegetable breeding company.

**Table 6:** The nutrient concentrations in the starter solution and within the nutrient solution supplied to the pepper plants throughout the vegetative and reproductive growth stages.

Nutrient	Starter Solution (13 November 2021)	Vegetative Phase (14 November 2021)	Reproductive Phase (7 February 2022)	Unit
NO_3_^−^	16.05	15.79	16.64	mM
K^+^	5.70	5.86	6.94	mM
Ca^2+^	6.15	5.60	5.55	mM
Mg^2+^	2.50	1.63	1.66	mM
SO_4_^2−^	3.27	2.10	2.08	mM
H_2_PO_4_^−^	1.25	1.35	1.35	mM
NH_4_^+^	1.05	1.22	1.00	mM
Fe	20.00	20.00	16.20	μM
Mn^2+^	12.00	12.00	10.80	μM
Zn^2+^	7.00	6.00	5.40	μM
B	50.00	32.00	32.40	μM
Cu^2+^	0.80	0.80	0.86	μM
Mo	0.60	0.60	0.54	μΜ
Cl^−^	0.40	0.40	0.40	μΜ

## Data Availability

The data are included in the manuscript.
